# Interactive Skin Display with Epidermal Stimuli Electrode

**DOI:** 10.1002/advs.201802351

**Published:** 2019-04-26

**Authors:** Eui Hyuk Kim, Hyowon Han, Seunggun Yu, Chanho Park, Gwangmook Kim, Beomjin Jeong, Seung Won Lee, Jong Sung Kim, Seokyeong Lee, Joohee Kim, Jang‐Ung Park, Wooyoung Shim, Cheolmin Park

**Affiliations:** ^1^ Department of Materials Science and Engineering Yonsei University 50 Yonsei‐ro, Seodaemun‐gu Seoul 03722 Republic of Korea; ^2^ Insulation Materials Research Center Korea Electrotechnology Research Institute Bulmosan‐ro 10‐gil 12, Seongsan‐gu Changwon‐si Gyeongsangnam‐do 51543 Korea

**Keywords:** direct pressure and conductance visualization, field induced alternating current operation, fingerprint electroluminescent images, skin conformal devices, wearable sensing displays

## Abstract

In addition to the demand for stimuli‐responsive sensors that can detect various vital signals in epidermal skin, the development of electronic skin displays that quantitatively detect and visualize various epidermal stimuli such as the temperature, sweat gland activity, and conductance simultaneously are of significant interest for emerging human‐interactive electronics used in health monitoring. Herein, a novel interactive skin display with epidermal stimuli electrode (ISDEE) allowing for the simultaneous sensing and display of multiple epidermal stimuli on a single device is presented. It is based on a simple two‐layer architecture on a topographically patterned elastomeric polymer composite with light‐emitting inorganic phosphors, upon which two electrodes are placed with a certain parallel gap. The ISDEE is directly mounted on human skin, which by itself serves as a field‐responsive floating electrode of the display operating under an alternating current (AC). The AC field exerted on the epidermal skin layer depends on the conductance of the skin, which can be modulated based on a variety of physiological skin factors, such as the temperature, sweat gland activity, and pressure. Conductance‐dependent field‐induced electroluminescence is achieved, giving rise to an on‐hand sensing display platform where a variety of human information can be directly sensed and visualized.

The development of electronic skin (E‐skin) capable of mimicking the human skin representatively, including the sensing of a variety of delicate physiological changes of the epidermal stimuli, is of significant interest as an important human–machine interface that can play a key role in the numerous biomedical applications of human‐activity monitoring and personal healthcare.^1^, ^2^, ^3^, ^4^ Besides the efforts for high performance sensors capable of detecting individual epidermal stimuli such as the pressure, strain, and shear, as well as the body temperature and sweat, great emphasis has been placed on to develop single platform multifunctional epidermal sensors where diverse stimuli were detected independently. Most of them were, however, built by combining individual sensors on a common test bed.^5^, ^6^, ^7^, ^8^, ^9^, ^10^ Furthermore, visualization of the epidermal stimuli while sensing it can further extend the usefulness of an E‐skin by offering novel functions, including not only shape and position recognition, but also dynamic visual monitoring of the stimuli. Numerous pixelated and nonpixelated interactive displays were demonstrated, based on a variety of optical elements including light‐emitting diodes (LEDs),^11^, ^12^, ^13^, ^14^, ^15^ and thermochromic,^16^ electrochromic,^17^, ^18^ and triboelectrification devices^19^, ^20^, ^21^ and alternating current (AC)‐driven electroluminescent (ACEL) devices.^22^, ^23^, ^24^, ^25^, ^26^, ^27^, ^28^, ^29^, ^30^, ^31^, ^32^, ^33^, ^34^ Most of the interactive displays were again developed by physically combining stimuli‐sensors with the aforementioned display elements. Even the ACEL platforms were limited to simultaneous detection and visualization of a certain individual stimulus of either pressure or temperature.^35^, ^36^, ^37^, ^38^ The development of an interactive sensing display capable of detecting and visualizing multiple epidermal stimuli in *a single device* rather than ones with the combination of the individual sensors and displays on a common platform is, therefore, in great demand. We envision that multifunctional physiological skin display can be accomplished when the epidermal layer itself acts as an interactive display by replacing one of electronic components of the device by human skin.

Herein, we demonstrate a simple but robust interactive skin display with epidermal‐stimuli electrode (ISDEE) capable of simultaneous sensing and display of multiple epidermal stimuli on a single device. Our ISDEE with a simple two‐layer architecture consists of a topographically patterned elastomeric polymer composite with light‐emitting inorganic phosphors upon which two electrodes are placed with a certain parallel gap. When an ISDEE is directly mounted on the human skin, the skin surface by itself serves as a field‐responsive floating electrode of the display working under an AC. The AC field exerted on the epidermal skin layer depends on the conductance of the skin, which can be modulated as a function of a variety of physiological skin factors, such as the temperature, sweat gland activity, and pressure. Conductance‐dependent field‐induced electroluminescence is achieved, giving rise to an on‐hand sensing display, in which we are able to directly sense and visualize multiple types of human information including the temperature, sweat gland activity, and pressure as well as fingerprint.

Our ISDEE was conveniently developed by fabricating an elastomeric poly(dimethyl siloxane) (PDMS) composite with periodic arrays of topological micropyramids in which light‐emitting inorganic ZnS:Cu microparticles are embedded with the alumina layer for their surface passivation, followed by the deposition of two parallel PEDOT:PSS electrodes on the composite, as shown in **Figure** **1**a (Figure S1, Supporting Information). A 40 µm‐thick ZnS:Cu/PDMS composite with topological pyramidal arrays was prepared by spin‐coating a mixture on a micropatterned silicon mold, followed by thermal crosslinking of the PDMS. The replicated PDMS pyramids were successfully fabricated with an area and height of 5 × 5 µm^2^ and 5 µm, respectively, as evidenced in the scanning electron microscope (SEM) results shown in Figure 1b. A cross‐sectional view of the composite clearly shows that ZnS:Cu particles of ≈30 µm in diameter are embedded in the PDMS matrix (Figure 1c and Figure S2, Supporting Information). We extensively examined the mechanical properties of a neat PDMS and the PDMS composites with ZnS:Cu particles (Figure S3, Supporting Information). As expected, a rubbery, elastic PDMS became stiff when adding ZnS:Cu particles, as shown in the stress–strain curves. The elastic moduli of the neat PDMS and composites determined from the slopes of the stress–strain curves increased with the increase of the ZnS:Cu particles in the composites. In addition, we confirmed that the strain‐at‐break values of the samples decreased with the ZnS:Cu particles. By conformally placing the composite with two parallel electrodes on human skin, the skin serves as a floating electrode, called an epidermal‐stimuli electrode (EE), which consists of epidermal and dermal layers sensitive to pressure, temperature, and sweat stimuli from either the external environment or physiological signals of the internal body, as schematically illustrated in Figure 1a. Our ISDEE is ready to detect and visualize a variety of human‐related stimuli when sealed with an adhesive tape, as shown in the photographs in Figure 1d and e.

**Figure 1:**
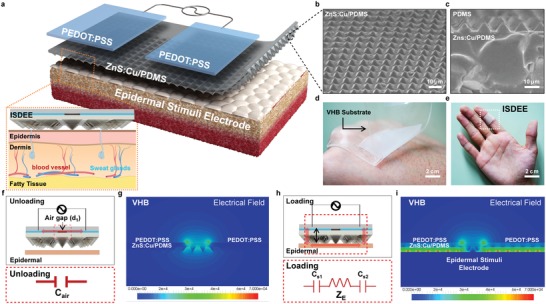
Device architecture and working principle of Interactive Skin Display with Epidermal Stimuli Electrode (ISDEE). a) Schematic of the device structure of parallel‐type PEDOT:PSS electrodes and a sensing layer with topological micropyramids consisting of a ZnS:Cu/PDMS composite on an epidermal stimuli electrode. b) An SEM image of a topological‐patterned ZnS:Cu/PDMS composite with micropyramids. c) A cross‐sectional SEM image of the composite with ZnS:Cu particles. Photographs of an ISDEE mounted on d) back of hand and e) finger. Schematic illustration and the equivalent electrical circuit model f,h) and FEA of AC field analysis g,i) of an ISDEE with distribution of electric field magnitude on epidermal stimuli electrode upon unloading and loading, respectively.

The AC field applied between the two parallel electrodes has little effect on the composite owing to the in‐plane electric field. When a composite with two parallel electrodes is placed on epidermal skin, which is naturally conductive, the skin serves as a floating electrode, allowing for a sharing of the electric field, giving rise to a vertically driven electric field developed in the two overlapped areas on the AC field between the two electrodes. Under these circumstances, our device can detect either a change in capacitance or impedance depending upon a variety of vital skin information. The device scheme and electronic circuit diagram in Figure 1f and h illustrates the equivalent circuit model of an ISDEE upon unloading and loading, in which one fixed capacitor (*C*_air_) indicates the initial capacitance between the two in‐plane PEDOT:PSS electrodes, whereas the variable one (*C*_s_) and epidermal resistor (*Z*_E_) describe the active capacitance with the area of the electronic‐epidermal contact (Figure S4, Supporting Information).

The significant benefit of our device lies in the fact that all epidermal stimuli are visualized in EL arising from solid‐state cathode luminescence of ZnS:Cu particles embedded in the PDMS, in addition to electrical detection. A change in either the capacitance or the impedance with epidermal stimuli can vary the electric field upon AC operation, giving rise to a change in EL intensity. The generation of an effective electrical field using the epidermal stimuli electrode of our ISDEE was confirmed through an electrical field calculation based on a finite element analysis (FEA), the results of which are shown in Figure 1g and i. The calculation shows that the electrical field built between the PEDOT:PSS electrodes before contact on the skin was significantly spread out and concentrated toward the two overlapped skin areas when a composite was conformally placed on the skin.

Prior to monitoring the pressure exerted on the human skin using our ISDEE, we examined the pressure‐sensing performance of a parallel‐type AC device with a floating indium tin oxide (ITO) electrode instead of skin, as shown schematically in **Figure** **2**a. The dielectric constants of ZnS:Cu/PDMS composites have little effect on frequency except the slight decrease at a high frequency regime, which was much higher than the typical operation frequency for a parallel AC device, as shown in Figure 2b. The presence of ZnS:Cu particles in the composites was also advantageous for the sensitive detection of pressure in the capacitance owing to the enhanced dielectric properties of a composite with semiconducting particles (Figure S5a and b, Supporting Information).

**Figure 2:**
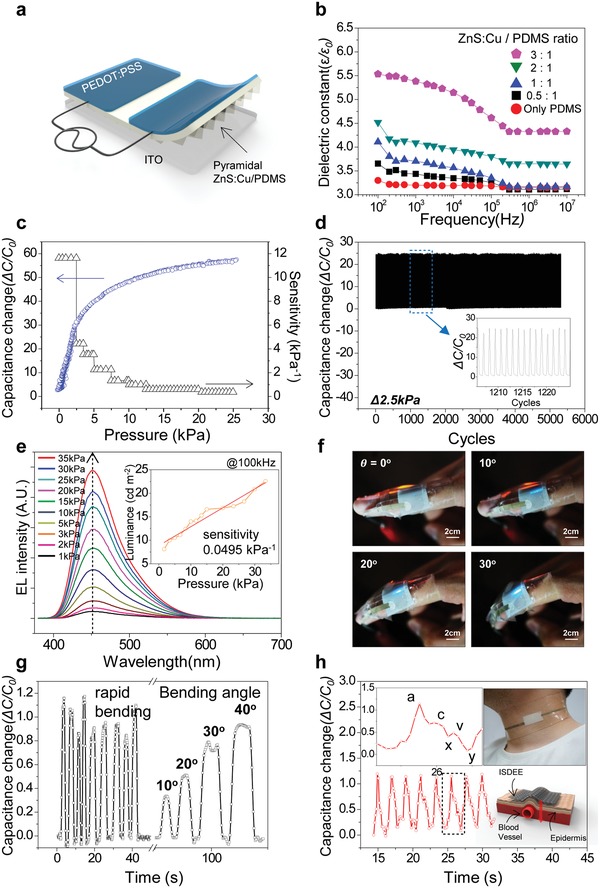
Properties of pressure‐sensing and visualization of a parallel‐type AC device. a) Schematic of a parallel‐type AC device with a floating ITO electrode. b) AC frequency‐dependent dielectric constant of ZnS:Cu/PDMS composite with different ratios. c) A plot of the change in capacitance and sensitivity as a function of pressure with a parallel‐type AC device with a composite ZnS:Cu/PDMS at a weight ratio of 3:1. The sensitivity is defined as *S*_c_ = δ(*∆C/C*_0_)/*δp*, where *p* is the applied pressure, and *C* and *C*_0_ are the capacitances with and without the applied pressure, respectively. d) Load–unload cycle endurance in capacitance changes over 5000 cycles at Δ2.5 kPa. e) EL intensity of the device under different applied pressures of 1 to 35 kPa. The inset shows a plot of the integrated EL luminescence as a function of pressure. The EL sensitivity is defined as S_EL_ = δ(*∆L/L_0_*)/*δp*, where *p* is the applied pressure, and *L* and *L*_0_ are the integrated EL intensities with and without applied pressure, respectively. f) Photographs of an ISDEE mounted on finger with EL upon finger motion as a function of the bending angle. g) Time‐dependent capacitance changes of the ISDEE attached to a finger for human motion sensing. h) Measurement of JVP patterns by the ISDEE attached to the middle of the neck‐packing VHB film. The device was operated at AC voltage and a frequency of 100 V and 100 kHz, respectively. The ISDEE also has a composite ZnS:Cu/PDMS (3:1 in weight ratio).

The advantage of our pressure sensor operated with a parallel AC field lies in its extremely low initial capacitance because such capacitance is regarded as the value obtained from the in‐plane capacitor of PEDOT:PSS/air/PEDOT:PSS right before contact, as schematically shown in Figure 1f (Figure S6, Supporting Information). When a composite with two parallel electrodes is contacted on a floating electrode, the capacitance is field‐driven and the second capacitance (*C*_s_) becomes dominant (Figure 1h), as determined using a vertical capacitor of the floating electrode/composite with a pyramidal air gap/PEDOT:PSS. When further pressurized, the capacitance increases owing to both a decrease in thickness and an increase in the dielectric constant of the composite arising from the reduction of the pyramidal air gap (Figure S7, Supporting Information). As a consequence, the pressure sensitivity of the device was much higher than that of a flat composite capacitor, as shown in Figure 2c (Figure S8, Supporting Information). The highest sensitivity of ≈12 kPa^−1^ was achieved in our parallel‐type AC device, much greater than the values obtained from the sensors with the similar topological structures.^39^, ^40^, ^41^ The fast capacitance response and relaxation times (≈100 ms) upon pressure was obtained (Figure S9, Supporting information). In addition, the device exhibited an excellent load–unload endurance of over 5000 cycles under a pressure of 2.5 kPa, as shown in Figure 2d. Furthermore, we examined the pressure sensing properties of an ISDEE containing a ZnS:Cu/PDMS (3/1) composite upon repetitive bending events with the bending radius of 10 mm. The performance of the ISDEE was rarely altered after 1000 bending cycles (Figure S10, Supporting Information).

The pressure exerted on a parallel‐type sensor was successfully visualized using field‐induced ACEL while detecting the pressure in capacitance mode. EL spectra of a parallel‐type device containing a blue‐emission layer were obtained as a function of pressure; the results in Figure 2e clearly shows that the intensity of the light emission increased with the applied pressure (Figure S11, Supporting Information). The plot inset of Figure 2e shows that the EL intensity increased linearly with pressure. The EL sensitivity of the ISDEE calculated from the slope of the plot was ≈0.0495 kPa^−1^. EL performance of the sensor was also examined as a function of the amount of ZnS:Cu microparticles as well as the frequency^31^, ^32^, ^33^, ^34^ (Figure S12, Supporting Information).

A bottom electrode was readily replaced with an epidermal stimuli electrode by patching a bilayered topological composite with two parallel PEDOT:PSS electrodes placed on human skin, giving rise to an ISDEE, as shown in Figure 2f. First, finger‐bending motion was monitored using an ISDEE attached to a finger knuckle, as shown in the photograph of Figure 2f. In addition, the compression exerted on the knuckle upon bending was monitored in terms of the capacitance in real time, and the location of the maximum compression was at the same time visualized in the EL, as shown in Figure 2g. Both the capacitance and EL were enhanced with the bending angle proportional to the compression (Figure S13 and Video S1, Supporting Information). Our ISDEE also allowed for monitoring the finger touch motion in terms of both the capacitance and EL intensity (Figure S14, Supporting Information). Furthermore, our ISDEE enabled us to detect human breath as well as a jugular venous pulse (JVP) signal for respiration (Figure 2h and Figure S15, Supporting Information). It should be, however, noted that, in spite of the capability of visualizing the pressure in EL over a broad range of pressure levels, the respiration modes involving delicate changes in pressure were difficult to visualize in EL mainly owing to the limit of the EL sensitivity of our ISDEE. The operation voltage we used for an ISDEE may be high to cause some damage on the skin. The electric field exerted on an ISDEE with a composite film of ≈30 µm in thickness was, however, 3.3 V µm^−1^ which is rarely harmful. We also monitored the current level of an ISDEE upon multiple contact events at the AC voltage and frequency of 100 V and 100 kHz, respectively (Figure S16, Supporting Information). The current of ≈2.4 mA was detected upon every contact event, corresponding to the unharmful current level. Because our ISDEE contains an epidermal stimuli electrode whose conductance can be varied with both body temperature and sweat, the information can be monitored in terms of the impedance change as long as the AC field exerted on the ISDEE is conductance‐dependent. To prove this, we examined how the sensing performance of a parallel‐type device is conductance‐dependent with a PEDOT:PSS floating electrode, whose conductance varies with the amount of dimethyl sulfoxide (DMSO) mixed with PEDOT:PSS.^35^ Impedance (*Z*) changes of parallel‐type AC devices with the floating PEDOT:PSS electrodes containing different amounts of DMSO were examined as a function of AC frequency and the results as shown in **Figure** **3**a. As expected, the impedance decreased with the conductance of the floating electrode as a function of DMSO concentration, representatively at a typical operation frequency of 100 kHz, as shown in Figure 3b. The conductance‐dependent ACEL performance of the devices was also observed using PEDOT:PSS electrodes containing different amounts of DMSO, as shown in Figure 3c (also see Figure S17, Supporting Information).

**Figure 3:**
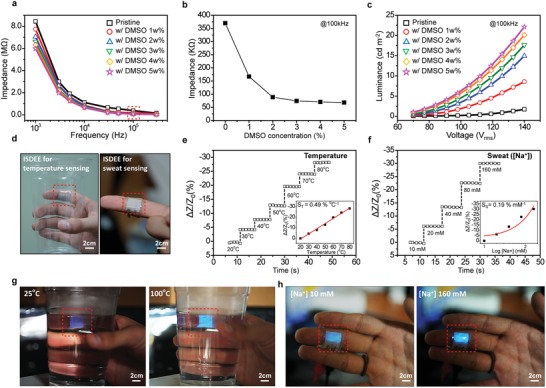
Properties of temperature and sweat sensing and visualization of an ISDEE. a) Variation in impedance (*Z*) of parallel‐type AC devices with floating PEDOT:PSS electrodes containing different amounts of DMSO as a function of frequency. b) A plot of impedance values of the devices at the frequency of 100 kHz as a function of DMSO in the PEDOT:PSS electrodes. c) Luminance versus voltage (L–V) characteristic of the parallel‐type AC devices with PEDOT:PSS top electrodes containing different amounts of DMSO. The device containing a topological‐patterned composite ZnS:Cu/PDMS at a weight ratio of 3:1 was operated at 100 kHz. d) Photographs of an ISDEE mounted on the skin for sensing temperature and sweat. The ISDEE containing a topological‐patterned composite ZnS:Cu/PDMS at a weight ratio of 3:1 was operated at AC voltage and a frequency of 100 V and 100 kHz, respectively. e) Time‐dependent variation of change in impedance and temperature immediately after release of the grasped cup on ISDEE with temperature detection. The sensitivity is defined as *S*_T_ = δ(*∆Z/Z_0_*)/*δT*, where *T* is the applied temperature, and *Z* and *Z*_0_ are the impedances with and without the applied temperature, respectively. f) Variation of the change in impedance as a function of sweat from human skin with a relative sweat concentration of 0–160 × 10^−3^ m. The sensitivity is defined as *S*_S_ = δ(*∆Z/Z_0_*)/*δC*, where *C* is the [Na^+^] concentration, and *Z* and *Z*_0_ are the impedances with different concentration. g) Photographs of EL intensity images with surface temperature 25 °C (left) and 100 °C (right). h) Photographs of EL intensity images with sweat concentration of 10 × 10^−3^ m (left) and 160 × 10^−3^ m (right).

The detection and visualization of the change in conductance of a floating electrode in terms of the impedance and EL, respectively, allowed us to utilize our ISDEE as a sensing display for both body temperature and sweat gland activity, which is sensitive to skin impedance under an AC field arising from the reorientation of lipids in the sweat glands underneath the epidermis depending upon the salt concentration in sweat. As a result, the skin impedance decreases with both body sweat and temperature. For the demonstration, an ISDEE was fabricated using a finger epidermal stimuli electrode, as shown in the photographs of Figure 3d. Figure 3e and f shows a linear response of the resistive impedance sensor within temperature range of 20–80 °C with a sensitivity of ≈0.49 % °C^−1^ and [Na^+^] concentrations in the electrolyte solutions with relevant concentrations of 10 × 10^−3^ –160 × 10^−3^ m with a sensitivity of ≈0.19% mm ^−1^, respectively. When the ISDEE was touched on the surface of a glass cup filled with hot water, the temperature of the water was transferred to the skin electrode, giving rise to a change in impedance as well as EL upon the AC field. The emissions became brighter with the surface temperature, as shown in Figure 3g. A change in sweat‐dependent impedance was also observed in our ISDEE, the results of which are shown in Figure 3h. When the ISDEE on a finger was in contact with skin under a high state of sweating ([Na^+^] 160 × 10^−3^ m), the impedance of the device was abruptly reduced, and the original value was recovered when untouched. Multiple sweat sensing was accomplished with reliability. In addition, the concentration of sweat can be clearly visualized using EL, as shown in Figure 3h. We examined the effect of the relative humidity on the impedance of an ISDEE (Figure S18, Supporting Information). When the impedance of an ISDEE was monitored with the relative humidity ranging from 30% to 80% at room temperature, the impedance was rarely varied with the relative humidity. The results imply that the epidermal electrode in an ISDEE with the humidity as high as ≈90% was hardly affected by the relative humidity of surrounding environment. It should be noted that in spite of the multifunctional sensing and display of pressure, temperature, and sweat with a single ISDEE, it was not trivial completely to avoid the coupling of two sensing performances. In fact, while capacitance sensing of pressure was rarely affected by either temperature or sweat, the impedance change utilized for either temperature or sweat sensing was influenced by the pressure (Figure S19, Supporting Information). Materials and device design for pressure‐independent impedance change is under investigation.

Furthermore, our ISDEE offers a useful way to achieve the direct imaging of a 2D conductive biological information when an epidermal stimuli electrode contains a 2D pattern, such as a naturally conductive fingerprint, as schematically shown in **Figure** **4**a. Our ISDEE for visualizing fingerprint patterns was mounted to a finger, as shown in the photograph in Figure 4b. When the finger with the ISDEE was gently touched on any transparent surface, a distinct fingerprint instantly appeared with high resolution. It is also apparent that efficient quantitative pressure sensing was achieved as a function of the fingertip pressure in terms of capacitance (Figure S20 and Video S2, Supporting Information). Notably, the topographic pyramidal arrays of a composite layer are beneficial for high‐resolution imaging of a fingerprint. Compared with the fingerprint images obtained from the ISDEE with a flat composite layer, the image from that using the pyramidal arrays shows very discrete valleys and ridges in the fingerprint. In particular, the image was barely resolved under a high pressure of the ISDEE with a flat composite layer, as shown in Figure 4c. Fingerprint imaging using our ISDEE was advantageous as compared with a conventional fingerprint reader because the fingerprint could be shown on any transparent surface, as the series of photographs in Figure 4d. In principle, fingerprint identification can be successfully achieved when an ISDEE is conveniently attached to a finger touching anywhere on a transparent device.

**Figure 4:**
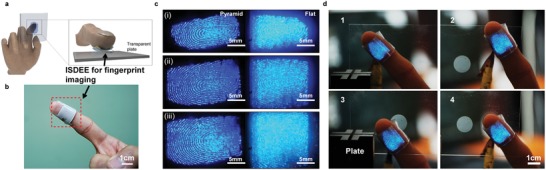
Imaging of high‐resolution fingerprint using an ISDEE. a) Schematic illustration of an ISDEE mounted on a fingertip and touched on a transparent substrate for visualizing a fingerprint in EL in addition to capacitance sensing of the touch. b) Photograph of an ISDEE mounted directly onto a finger. c) Photographs of EL images of fingerprint patterns obtained through conformal contact of a finger with an ISDEE with different pressure. The images are shown from ISDEEs with the microstructured (left column) and flat (right column) ZnS:Cu/PDMS (3:1 in weight ratio) composite layers. d) Photographs of an ISDEE with a microstructured ZnS:Cu/PDMS (3:1 in weight ratio) composite layer mounted on a finger, subsequently touched on arbitrary places on a transparent plate, giving rise to distinct fingerprint EL images on the touched areas. All ISDEEs were operated at AC voltage and a frequency of 80 V and 100 kHz, respectively.

In summary, this study presented a novel single device, ISDEE capable of visualizing a variety of body information such as touch, body temperature, and sweat with the simultaneous sensing of either the capacitance or impedance. The ISDEE was developed using a topographically surface‐structured elastomeric PDMS composite containing light‐emitting ZnS:Cu particles with two parallel PEDOT:PSS electrodes attached on a normal epidermal skin surface. The utilization of an epidermal skin layer as one of electrodes not only simplifies the device architecture (a bilayered structure), but also is capable of both sensing and displaying sensitive. First, the device visualized tactile sensations and finger‐bending motion in EL under an AC field while detecting such elements based on a change in capacitance. Furthermore, both body temperature and sweat sensitive to the conductance of the epidermal‐stimuli electrode were monitored based on changes in AC impedance through a direct visualization in EL. A single ISDEE exhibited the multiple sensing performance of 0.49 % °C^−1^, 0.19 % mM^−1^, and 11.63 kPa^−1^ for sensing temperature, sweat gland activity, and pressure, respectively, as well as a direct visualization of these epidermal stimuli. The ISDEE also allows for direct imaging of fingerprint patterns with a high resolution while achieving sensitive capacitance detection. The suitability of our extremely simple, but multimode, multifunctional skin display platform with high sensing performance (Table S1, Supporting Information) can be expanded for numerous emerging biomedical applications of human‐activity and health‐monitoring systems.

## Experimental Section

*Materials*: Green ZnS:Cu microparticles (D512C) were purchased from Shanghai KPT Co. PDMS and crosslinkers were purchased from Dow Corning. The PEDOT:PSS electrode (Clevios PH1000) was modified through mixing with 5 wt% DMSO and 1 wt% Zonyl surfactant (FS‐300 fluorosurfactant from Aldrich) with respect to PEDOT:PSS, which promoted the wetting of the ZnS:Cu/PDMS composite layer on an active layer. The 3M VHB tape (3M, VHB 4905) used was purchased and applied as received. Trichloro(1H,1H,2H,2H‐perfluorooctyl)silane (FOTS) was purchased from Sigma‐Aldrich. All other materials were purchased from Aldrich and also used as received.

*Fabrication of an ISDEE*: An interactive sensing display was developed using a parallel‐type AC device architecture, as illustrated in Figure 1a. First, a micropatterned pyramidal relief mold was fabricated on a 4‐inch silicon wafer (using 300 nm thick thermally grown silicon oxide) using photolithography, followed by chemical etching. The arrays of the engraved pyramids were developed under P4mm symmetry, with the base area and height of each pyramid being 5 × 5 µm^2^ and 5 µm, respectively. The micropatterned Si mold was treated by O_2_ plasma at 40 W for 3 min before deposition of a FOTS solution, which facilitated the removal of a ZnS:Cu/PDMS composite layer from the Si mold. The ZnS:Cu/PDMS composite was prepared by mixing the ZnS:Cu powder with a PDMS liquid and a curing agent (Sylgard 184) with a weight ratio of 10:1. A liquid solution was spin‐coated on the micropatterned Si substrates at 2000 rpm for 120 s, and subsequently annealed at 80 °C for 12 h, followed by UV treatment for 20 min. High‐conductivity PEDOT:PSS was modified by mixing it with 5 wt% DMSO and a 1 wt% Zonyl surfactant with respect to PEDOT:PSS. A PEDOT:PSS layer of the modified solution was then spin‐coated onto the composite film. An ≈200 nm thick PEDOT:PSS film was subsequently annealed at 100 °C for 15 min in an ambient atmosphere. In addition, the conductive PEDOT:PSS layer was treated as separate electrodes with an air gap using reactive‐ion etching (RIE). Both the composite and PEDOT:PSS layers were peeled off from the Si molds and mechanically transferred onto a 3M VHB film.

*An ISDEE characterization*: The cross‐sectional morphology and thickness of the ZnS:Cu/PDMS composites were characterized using a field‐emission scanning electron microscope (FESEM) (JEOL‐7800F), as shown in Figure 1c. The capacitance and impedance were applied using a precision inductance, capacitance, and resistance (LCR) meter (Agilent E4980A). The frequency was varied from 100 Hz to 300 kHz. For changes in the measurement of the capacitance and impedance as a function of pressure and conductance, respectively, a computer‐controlled universal manipulator (Teraleader) was set up along with the LCR meter. The vertical spatial and force resolutions of the equipment are 1 µm and 10 mN, respectively. The luminance and EL spectra of the devices were obtained using a spectroradiometer (Konica CS 2000). A function generator (Agilent 33220A) connected with a high‐voltage amplifier (TREK 623B) was used for EL driving of the ISDEE. The current–voltage–luminance (I–V–L) characteristics of the devices were measured using a multichannel precision AC power analyzer (Zimmer Electronics Systems LMG 500). All measurements were conducted in a dark box under ambient conditions in air. Informed consent was obtained from the volunteer, who is one of the authors.

## Conflict of Interest

The authors declare no conflict of interest.

## Supporting information

Supplementary

Supplementary

Supplementary
