# Supplementary material for: Interactive Skin Display with Epidermal Stimuli Electrode
Source: Adv Sci (Weinh). 2019 Apr 26;6(13):1802351. doi: 10.1002/advs.201802351 (PMC6662062; doi:10.1002/advs.201802351)
Supplement: Supplementary file 1 — Supplementary [file ADVS-6-1802351-s002.pdf]

Copyright WILEY-VCH Verlag GmbH & Co. KGaA, 69469 Weinheim, Germany, 2019.

ADVANCED SCIENCE Open Access

Supporting Information

for *Adv. Sci.,* DOI: 10.1002/advs.201802351

Interactive Skin Display with Epidermal Stimuli Electrode

*Eui Hyuk Kim, Hyowon Han, Seunggun Yu, Chanho Park, Gwangmook Kim, Beomjin Jeong, Seung Won Lee, Jong Sung Kim, Seokyeong Lee, Joohee Kim, Jang-Ung Park, Wooyoung Shim, and Cheolmin Park**

Copyright WILEY-VCH Verlag GmbH & Co. KGaA, 69469 Weinheim, Germany, 2019.

Supporting Information

Interactive Skin Display with Epidermal Stimuli Electrode

*Eui Hyuk Kim[1], Hyowon Han[1], Seunggun Yu[2], Chanho Park[1], Gwangmook Kim[1], Beomjin Jeong[1], Seung Won Lee[1], Jong Sung Kim[1], Seokyeong Lee[1], Joohee Kim[1], Jang-Ung Park[1], Wooyoung Shim[1] & Cheolmin Park[1]**

[1]Department of Materials Science and Engineering, Yonsei University, Seoul 120-749, Korea.
[2]Insulation Materials Research Center, Korea Electrotechnology Research Institute, Bulmosan-ro 10-gil 12, Seongsan-gu, Changwon-si, Gyeongsangnam-do, 51543, Korea.

Prof. Cheolmin Park, Eui Hyuk Kim, Hyowon Han, Dr. Seunggun Yu, Chanho Park, Gwangmook Kim, Beomjin Jeong, Seung Won Lee, Jong Sung Kim, Seokyeong Lee, Joohee Kim, Prof. Jang-Ung Park, Prof. Wooyoung Shim: Department of Materials Science and Engineering, Yonsei University, 50 Yonsei-ro, Seodaemun-gu, Seoul, 03722 (Republic of Korea)
E-mail: cmpark@yonsei.ac.kr

Keywords: Wearable sensing display, Skin conformal device, Direct pressure and conductance visualization, Fingerprint electroluminescent image, Field induced alternating current operation

S1. Fabrication process of an ISDEE

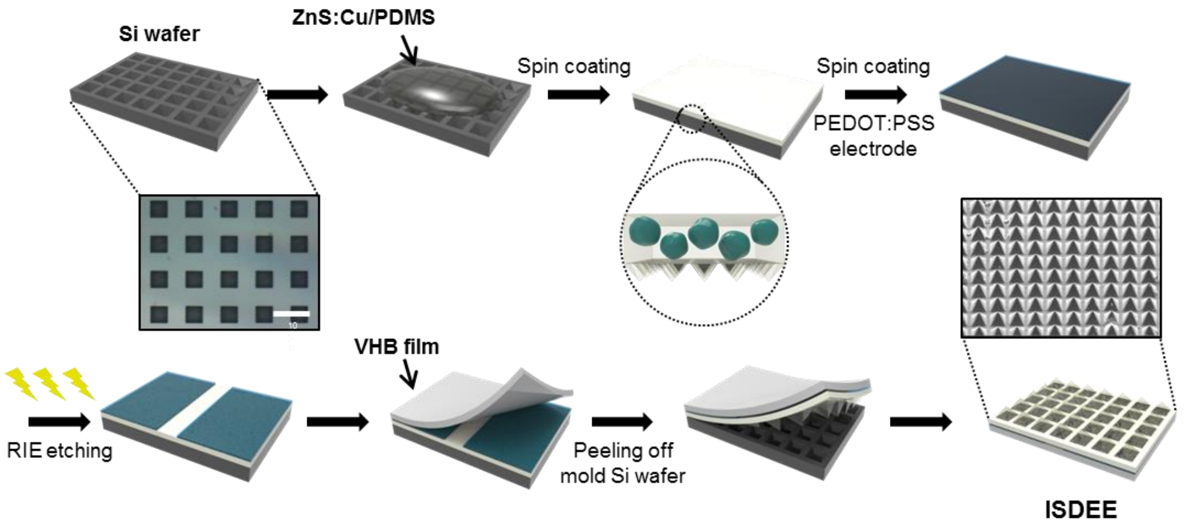

Figure S1. Fabrication processes of an ISDEE consisting of stacked bilayers. Schematic illustration of fabricating a topographically patterned ZnS:Cu/PDMS composite on a pre-patterned Si mold, followed by the deposition of in-plane PEDOT:PSS electrodes. The gap between two PEDOT:PSS electrodes was developed by RIE with a shadow mask. A VHB adhesive film was employed to remove the bilayers from the Si mold.

S2. A cross-sectional morphologies of a ZnS:Cu/PDMS composite layer

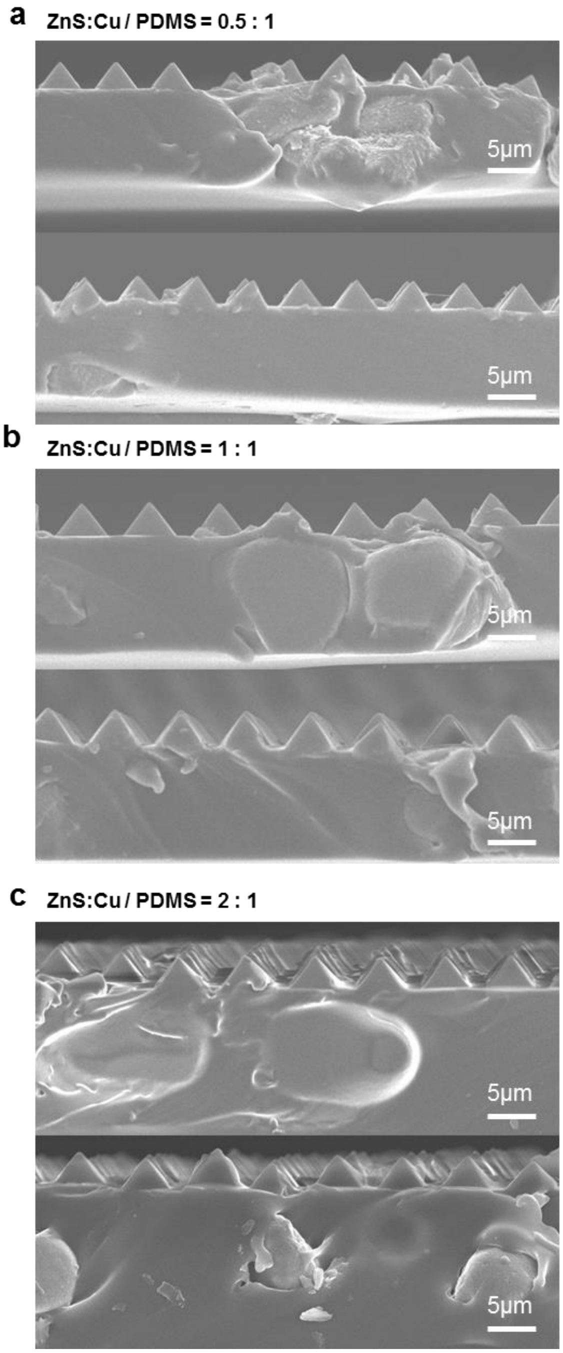

Figure S2. The cross-sectional scanning electron microscopy (SEM) images of ZnS:Cu/PDMS composites with various concentration of ZnS:Cu microparticles of (**a**) 50 wt%, (**b**) 100 wt% and (**c**) 200 wt% with respect to PDMS.

S3. The mechanical properties of a ZnS:Cu/PDMS composite

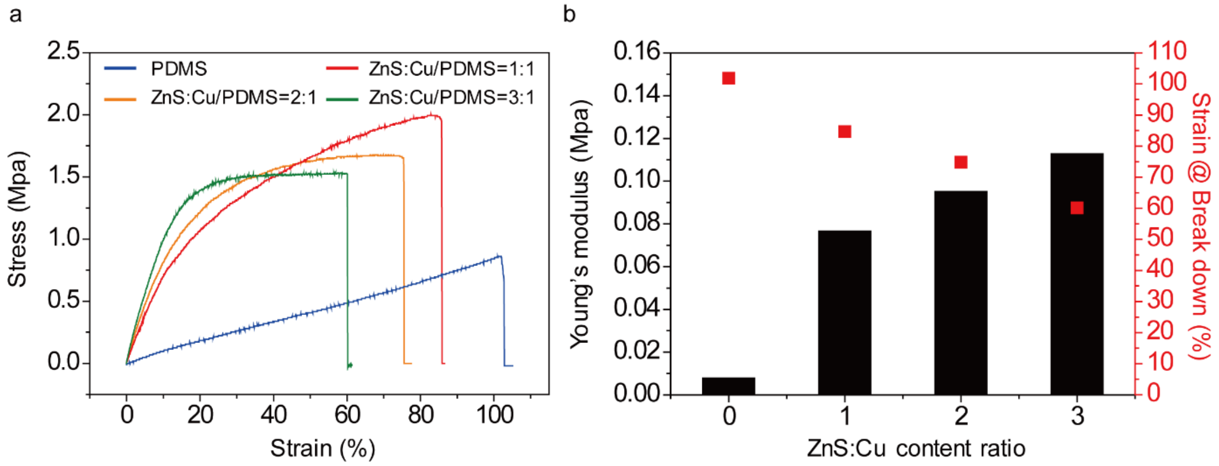

Figure S3. (a) The stress-strain curves of a neat PDMS and PDMS composites with different concentrations of ZnS:Cu particles. (b) Plots of the Young's moduli and the maximum strain-at-break values as a function of the ratios of ZnS:Cu particles to PDMS.

S4. Capacitive structure of a parallel-type AC device

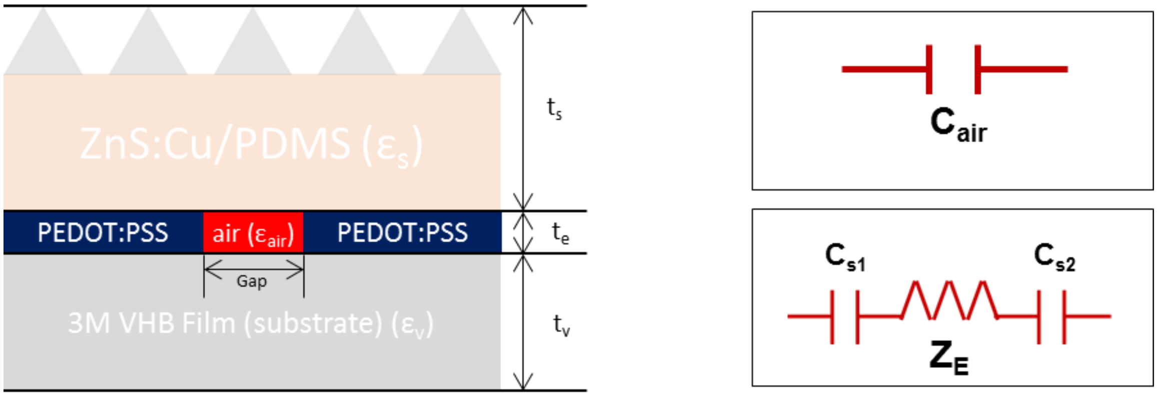

$$C_{s1} + C_{s2} = \varepsilon_0 \frac{\varepsilon_v + \varepsilon_s}{2} \frac{K\left[\sqrt{1 - \sqrt{\left(\frac{G}{2W+G}\right)^2}}\right]}{K\left[\frac{G}{2W+G}\right]}$$

$$C_{air} = \varepsilon_0 \varepsilon_{air} \frac{t_e}{G}$$

$$C_0 = C_{s1} + C_{s2} + C_{air}$$

Figure S4. Capacitive device structure of a parallel-type AC device with relative permittivity values: schematic, circuit and mathematical description of total capacitance, C_0 in terms of component capacitances of C_{s1}, C_{s2} and C_{air} with a number of interlocking "digits" of electrode width W, gap spacing G, and permittivity K.

S5. Optimization of the pressure sensing performance of a parallel-type AC device with floating ITO electrode

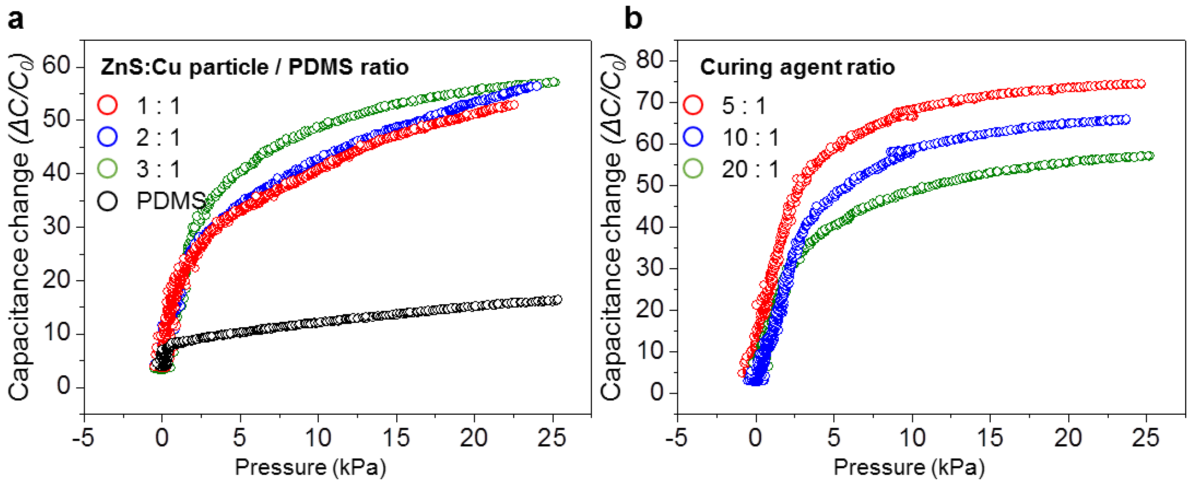

Figure S5. Capacitance change as functions of pressure of a parallel-type AC device with various concentration of (**a**) ZnS:Cu microparticles and (**b**) curing agent of PDMS.

S6-10. Operation mechanism and pressure sensing performance of parallel-type AC devices with floating ITO electrodes

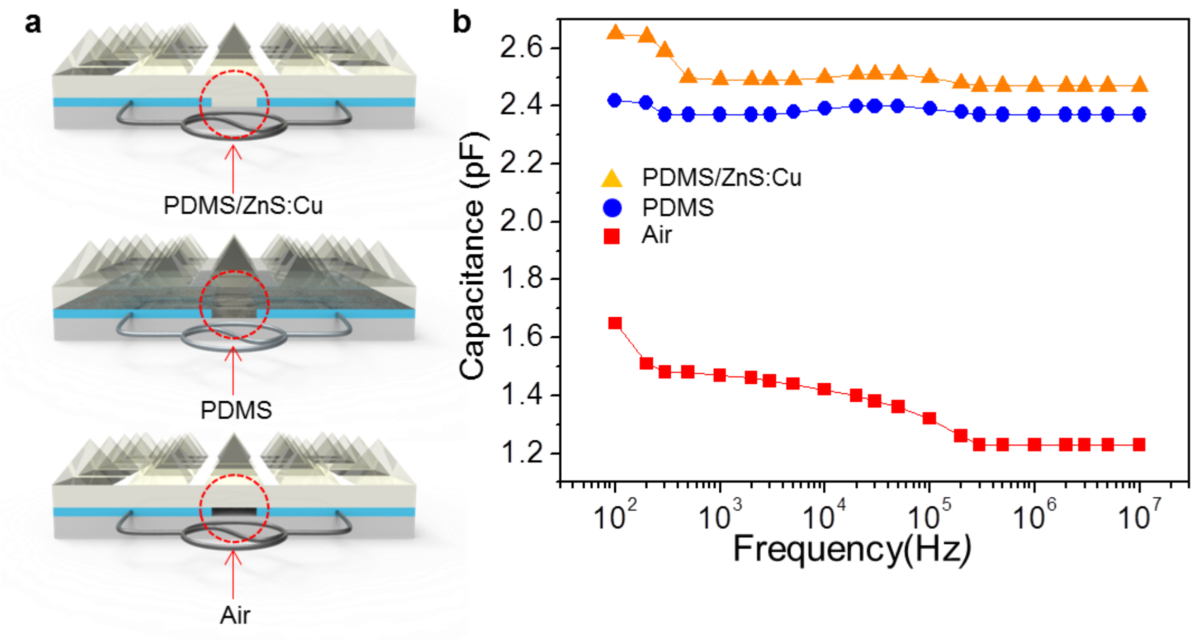

Figure S6. (**a**) Schematic of parallel-type AC devices with floating ITO electrodes which have different device structures between two in-plane PEDOT:PSS electrodes. The gaps between the two PEDOT:PSS electrodes were filled with ZnS:Cu/PDMS, PDMS and air from top to bottom. (**b**) Initial capacitance (C_0) as functions of AC frequency of the devices with different filled materials.

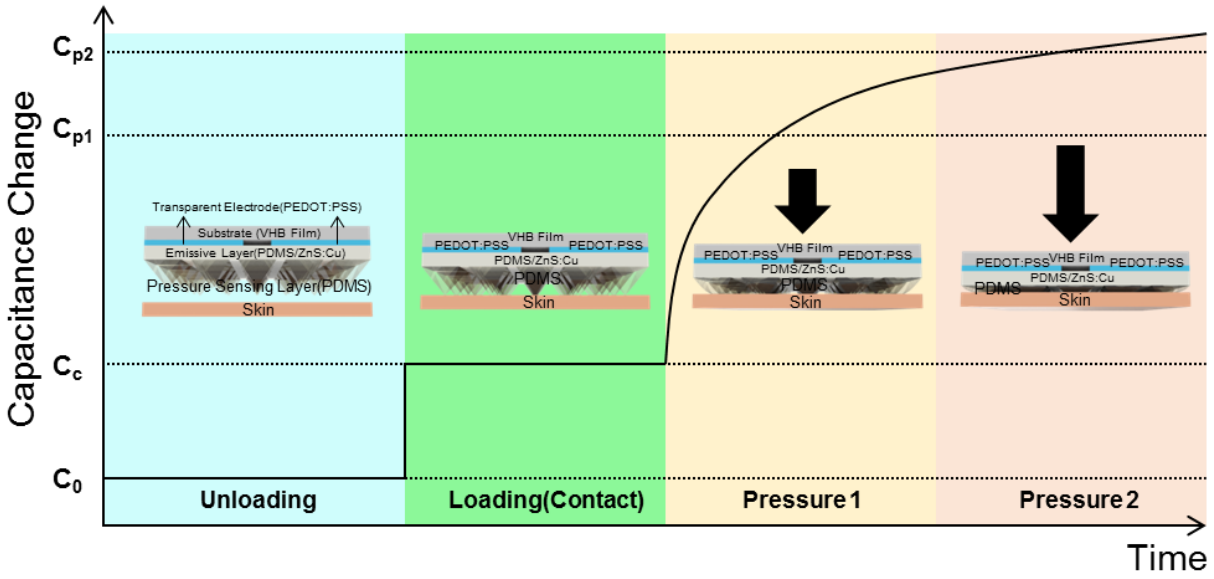

Figure S7. Operation mechanism of a parallel-type AC device with a floating ITO electrode based on a parallel capacitor device model. The initial capacitance (C_0) between two separated bottom electrodes without contact of ITO electrode. The capacitance of contact (C_c) is field-driven by ITO electrode on the device. The capacitance at pressure 1 (C_{p1}) and pressure 2 (C_{p2}) is increased, significantly reducing the air gap between the ITO and PEDOT:PSS electrode due to deformation of pyramids of the composite.

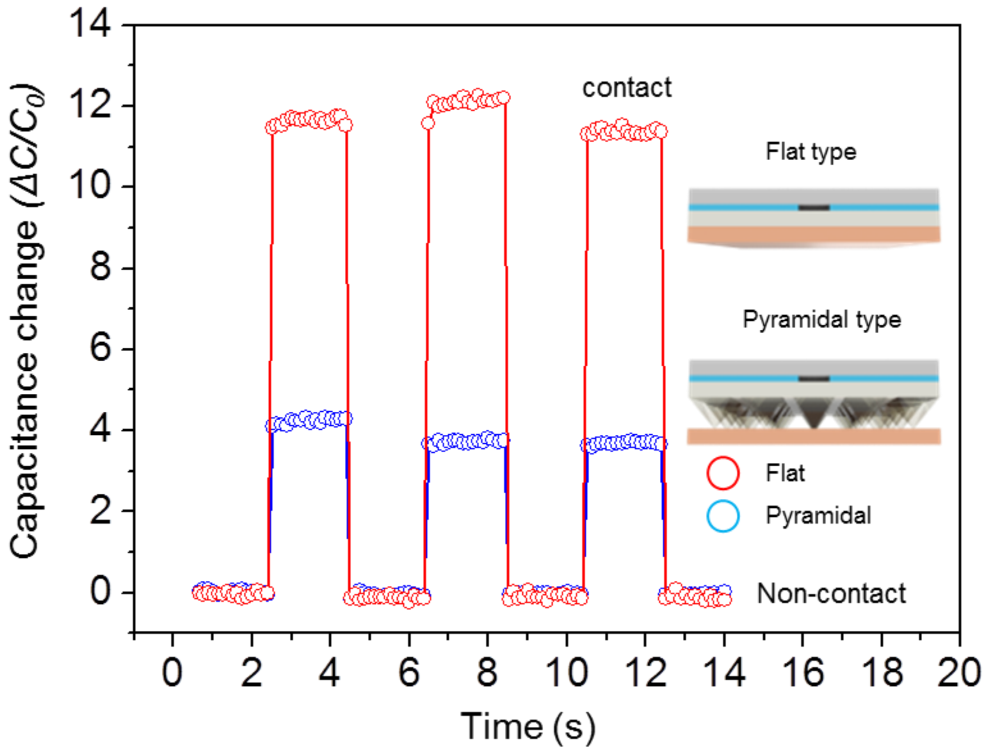

Figure S8. Capacitance change ($\Delta C/C_0$) of parallel-type AC devices containing flat and topological pyramidal type ZnS:Cu/PDMS composite layers as the pressure of 0.15 kPa was applied on the devices.

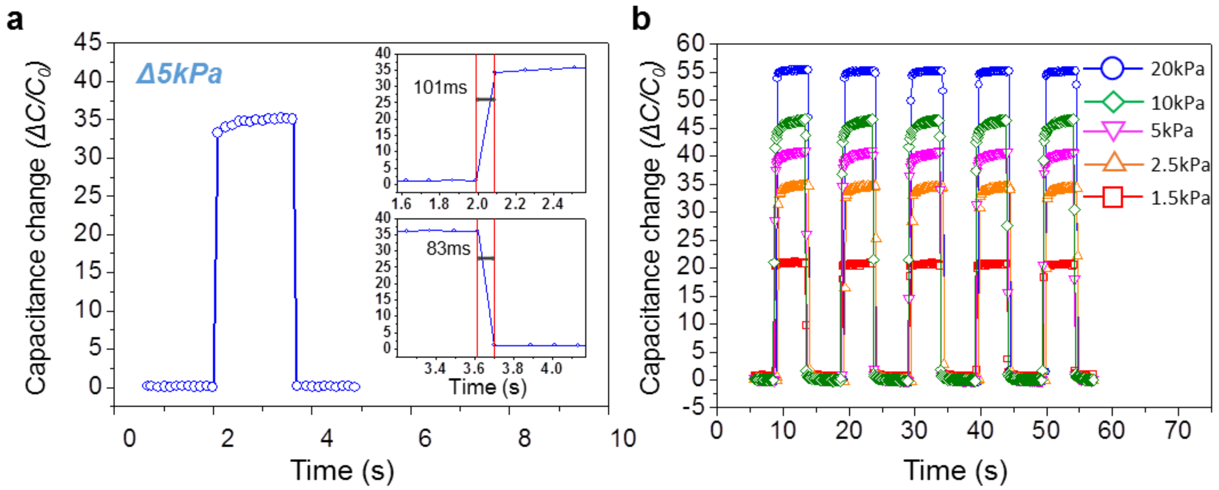

Figure S9. (**a**) Time-resolved capacitance change response of a parallel-type AC device with a floating ITO electrode at Δ5 kPa. Both response and relaxation of the capacitance occurred within 100 ms, as shown in the inset. (**b**) Time-resolved capacitance change response of the device under repeated mechanical loads, with different pressures ranging from 1.5 to 20 kPa.

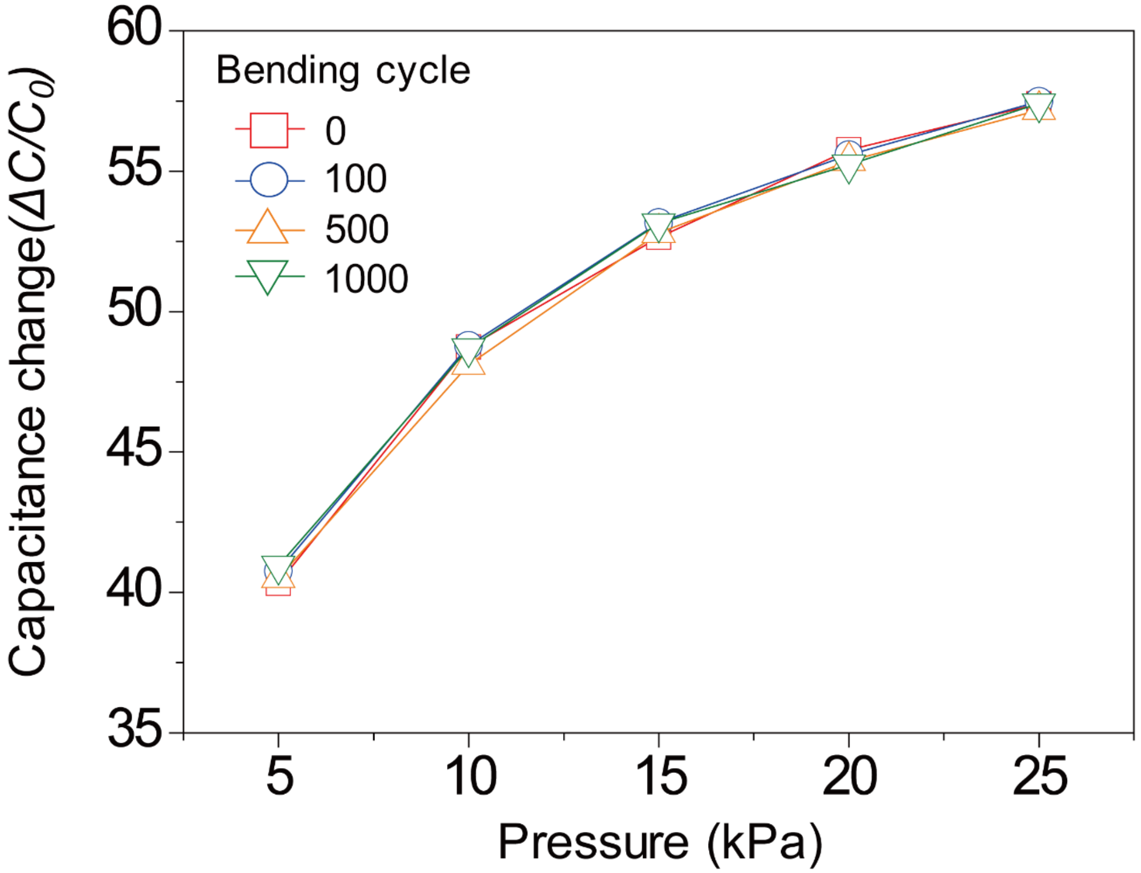

Figure S10. Capacitance change of an ISDEE containing a ZnS:Cu/PDMS (3/1) composite upon repetitive bending events with the bending radius of 10 mm.

S11-12. EL performance of parallel-type AC devices with floating ITO electrodes

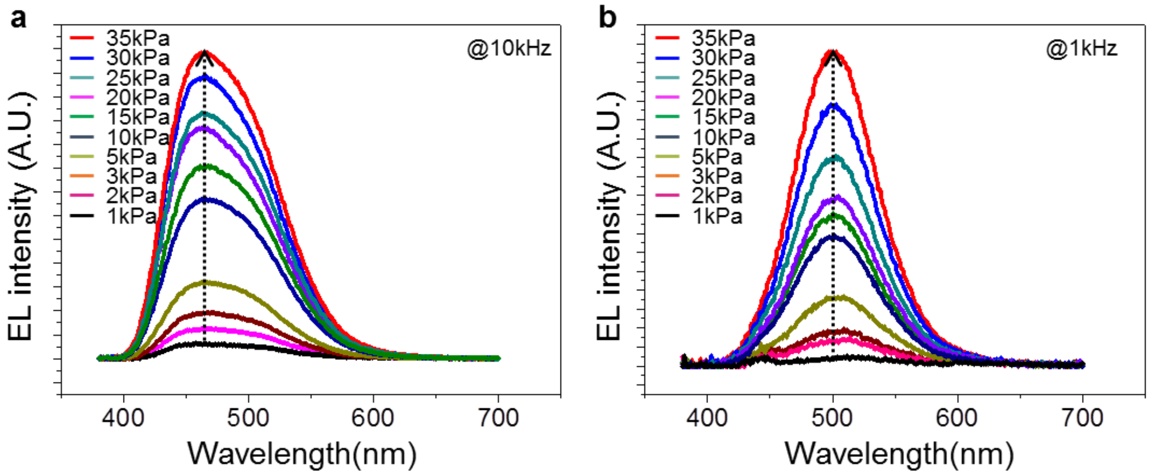

Figure S11. EL spectra of a parallel-type AC device with a floating ITO electrode as a function of applied pressures from 1 to 35 kPa at (**a**) 10 kHz and (**b**) 1 kHz.

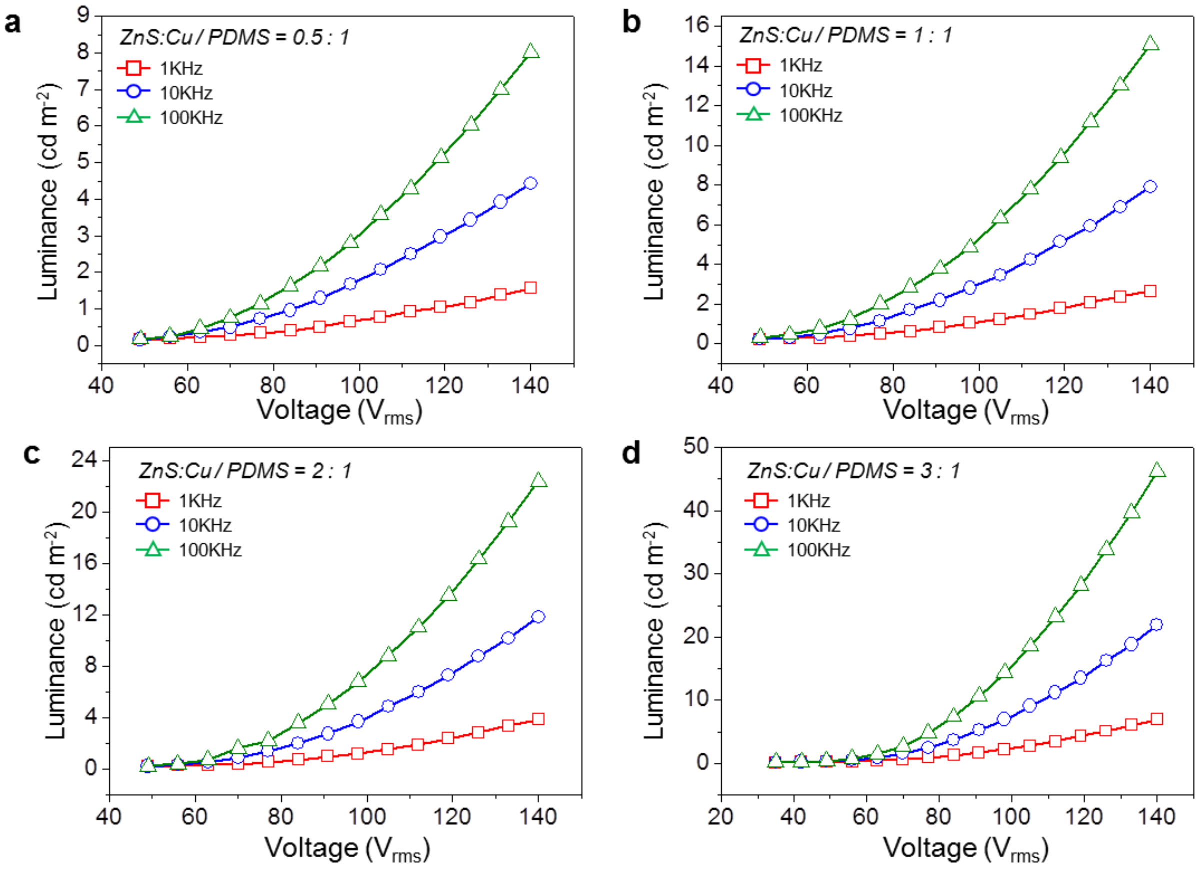

Figure S12. Luminance versus voltage (L-V) characteristics of parallel-type AC devices with floating ITO electrodes containing ZnS:Cu/PDMS composite layers having different ZnS:Cu contents of (**a**) 50 wt%, (**b**) 100 wt%, (**c**) 200 wt% and (**d**) 300 wt% with respect to PDMS. The device performance was examined at the three frequencies of 1, 10 and 100 kHz.

S13-14. EL performance of an ISDEE upon various mechanical motions

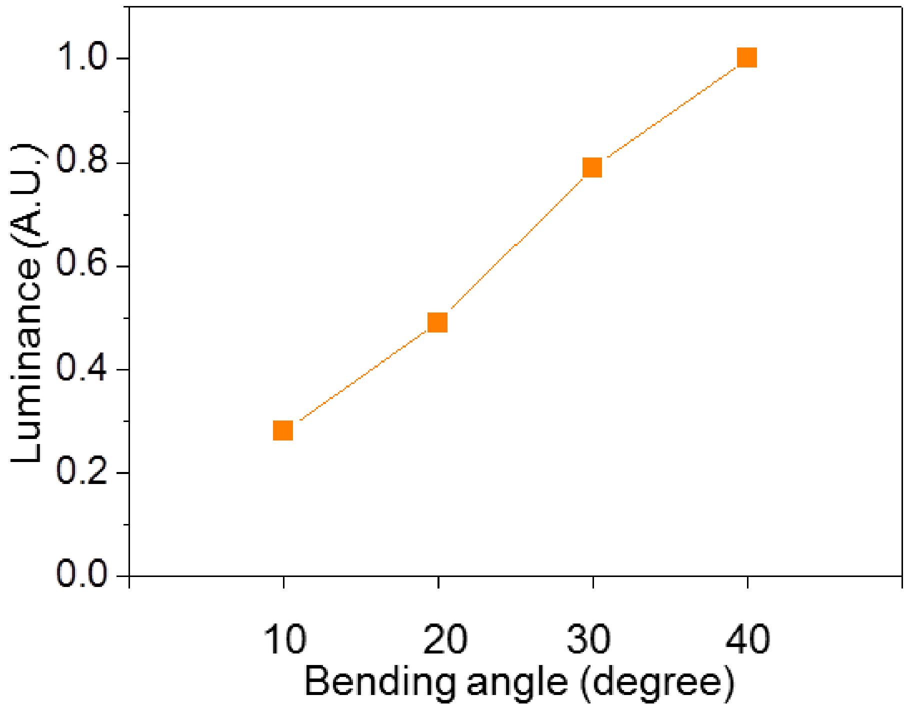

Figure S13. A plot of normalized EL intensity of an ISDEE as a function of bending angle.

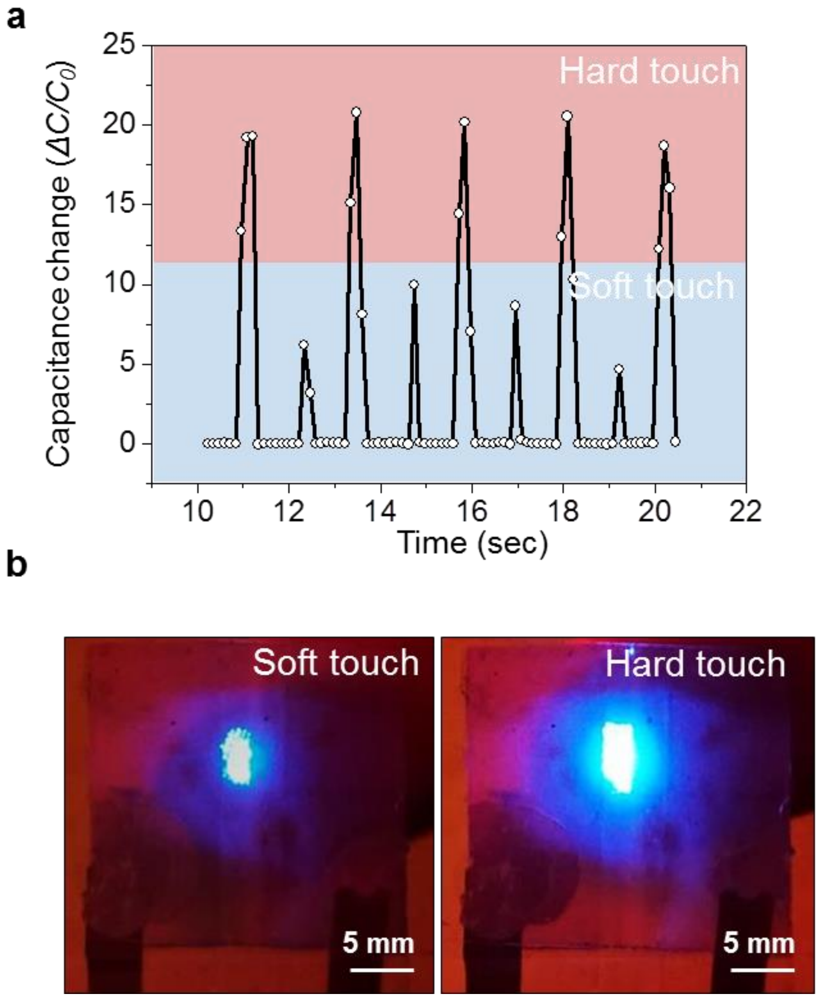

Figure S14. (**a**) Representative capacitive response of an ISDEE upon consecutive soft and hard touch events. (**b**) Photographs of the ISDEE showing strong EL upon the soft and hard touches.

S15. Respiration sensing of three distinctive breath modes with an ISDEE

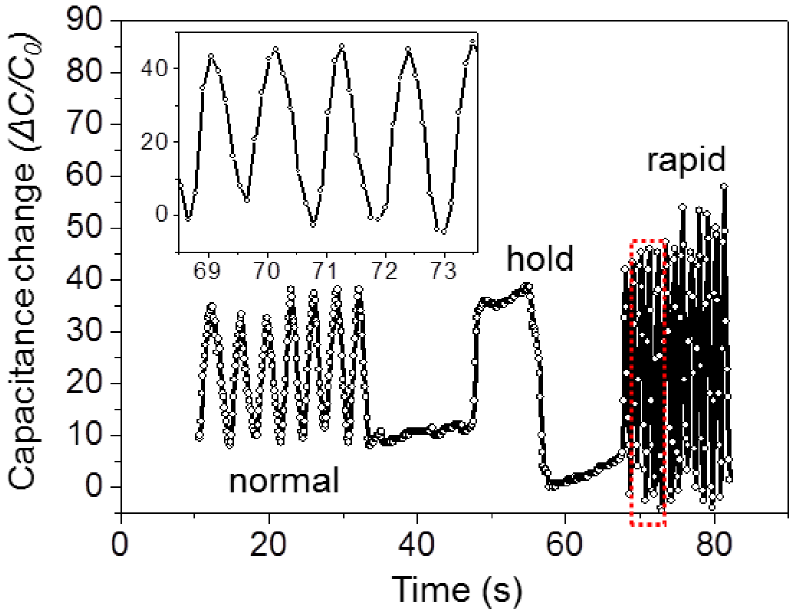

Figure S15. Time-dependent capacitance changes of breath signals recorded by an ISDEE mounted on the skin of the abdomen.

S16. AC current variation of an ISDEE upon repetitive skin contact

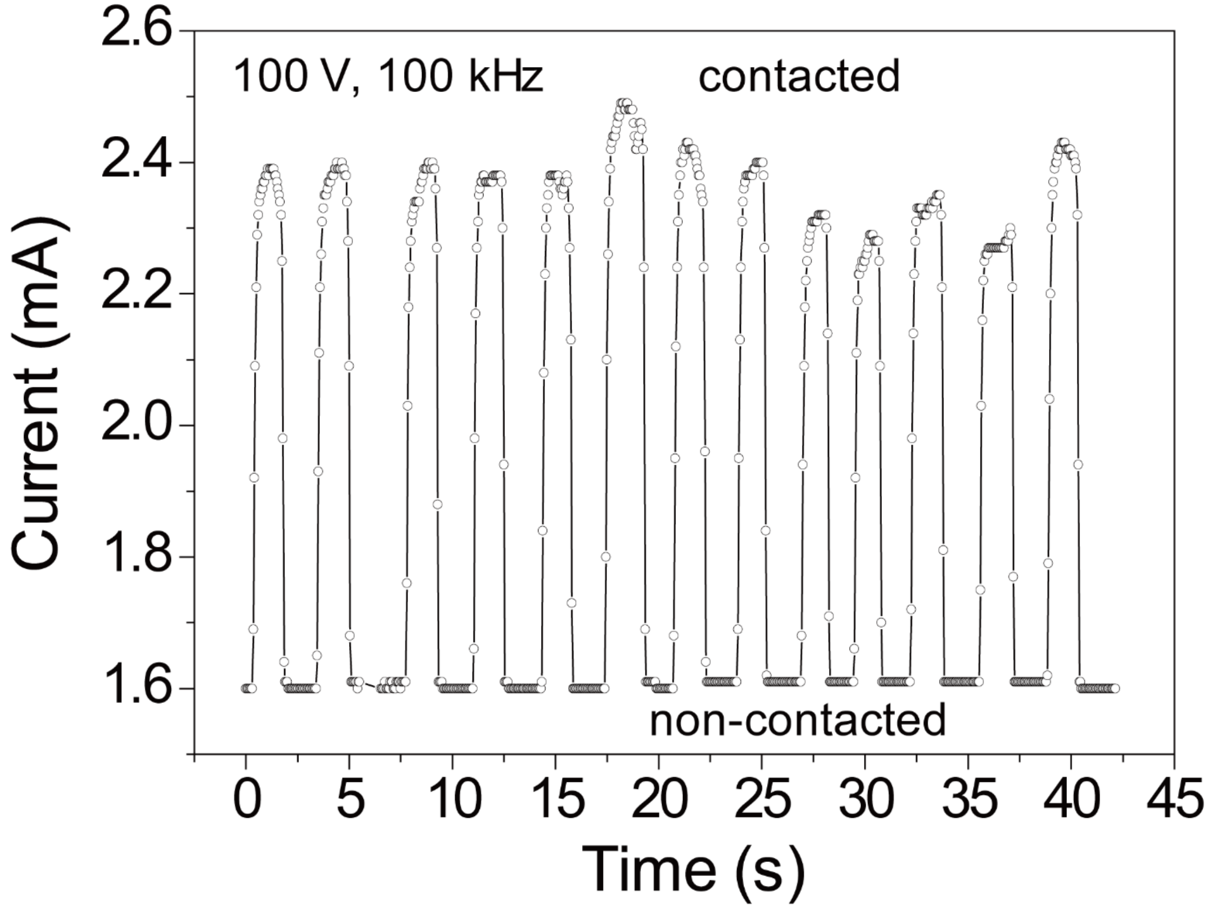

Figure S16. The current variation of an ISDEE under repetitive skin-contact and non-contact events at AC voltage and frequency of 100 V and 100 kHz, respectively.

S17. EL performance of a parallel-type AC device with floating PEDOT:PSS electrodes

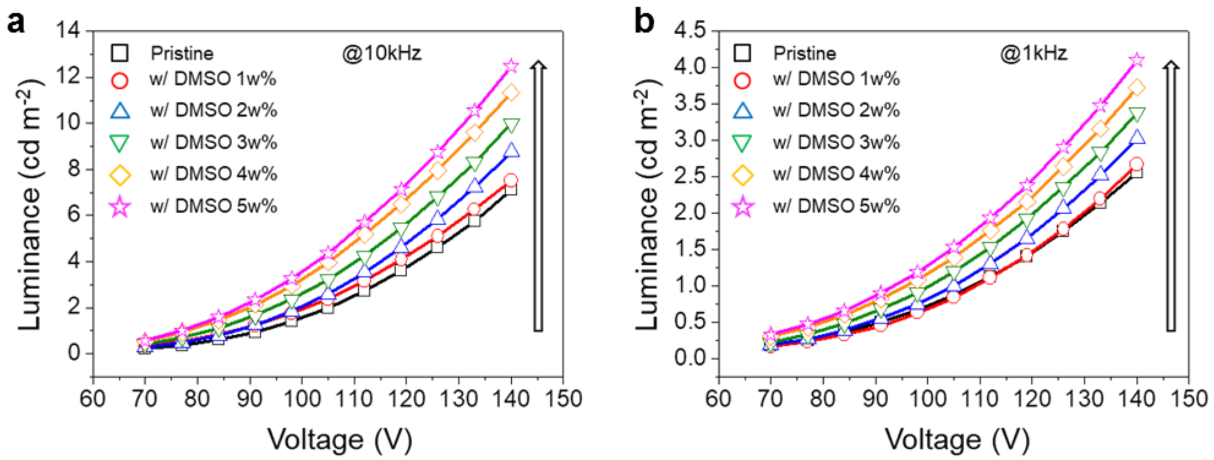

Figure S17. Luminance versus voltage (L–V) characteristic of parallel-type AC device with floating PEDOT:PSS electrodes containing different amount of DMSO operated at the AC frequency of (**a**) 10 kHz and (**b**) 1 kHz.

S18. The effect of humidity on impedance sensing performanc of an ISDEE

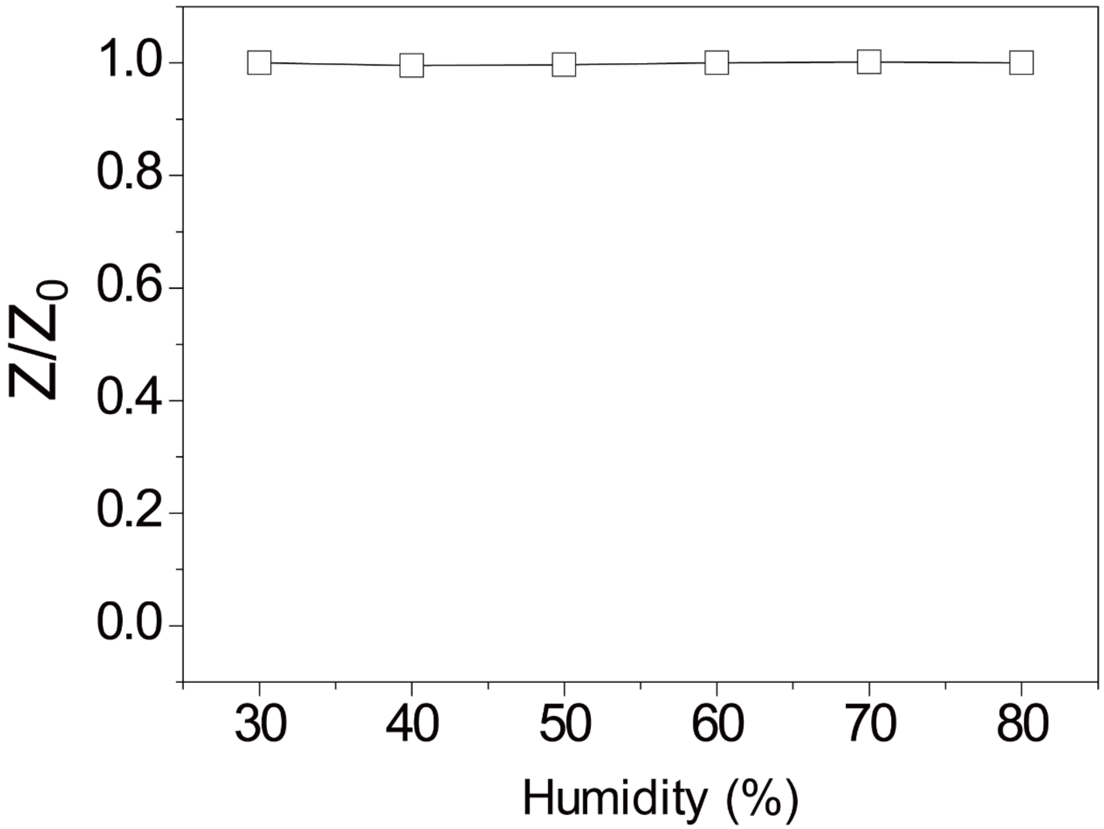

Figure S18. Variation in impedance of an ISDEE as a function of the relative humidity at room temperature.

S19. Simultaneous sensing of pressure and temperature with on the ISDEE

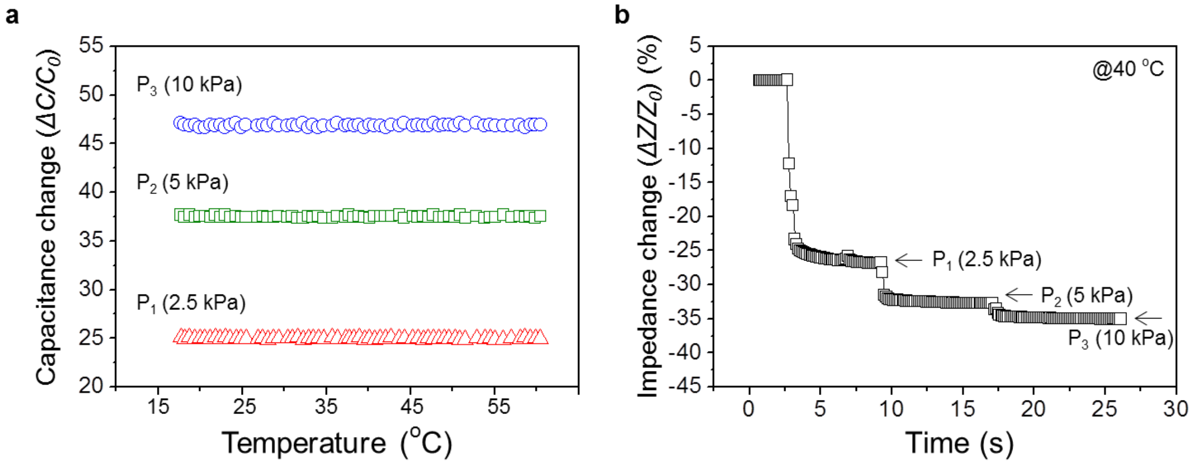

Figure S19. (**a**) A plot of the change in capacitance as a function of temperature with different pressure. (**b**) Time-dependent variation of change in impedance as a function of pressure at 40 °C.

S20. Pressure sensing performance of an ISDEE mounted on fingertip for simultaneous fingerprint imaging

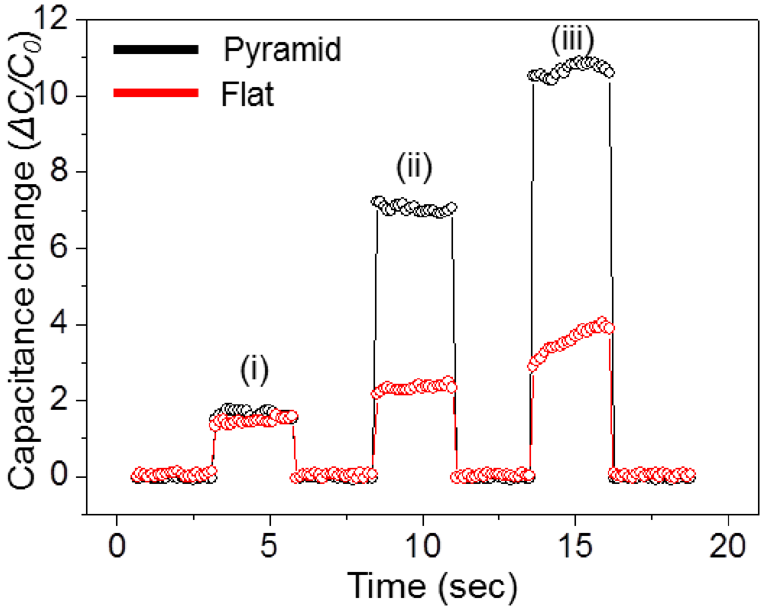

Figure S20. Variation of the change in capacitance as a function of pressure of a finger with an ISDEE. ISDEEs were examined with flat and topological-pyramidal ZnS:Cu/PDMS composite layers.

Type	Active materials	Response time	Sensing range	Sensitivity	Display (device or material)	Working mechanism	Ref.
Pressure	CNT fiber/Ecoflex	~ 63 ms	0.38 pa to 25 kPa	0.034-0.05 kPa^{-1}	X	Capacitance	1
	AgNW/PDMS	~ 40 ms	<1.4 MPa	1.62 MPa^{-1}	X	Capacitance	2
	AgNP-SBS composite/PDMS	~ 40 ms	< 20 kPa	0.21 kPa^{-1}	X	Capacitance	3
	P(VDF-TrFE-CFE)/[EMI][TFSA]	~ 25 ms	< 140 kPa	12 kPa^{-1}	O (ACEL)	Capacitance	4
	SWCNT/PDMS	~ 10 ms	0.6 pa to 1.2 kPa	1.8 kPa^{-1}	X	Resistance	5
	CNT/PDMS with microdome	~ 40 ms	0.2 pa to 59 kPa	15.1 kPa^{-1}	X	Resistance	6
	rGO/PU	-	< 10 kPa	0.26 kPa^{-1}	X	Resistance	7
	Pressure-sensitive rubber (PSR)	~ 1 ms	1 kPa to 98 kPa	-	O (LED)	Resistance	8
	AgNW/PU	-	-	-	O (Perovskite)	Resistance	9
	Carbon black/PDMS	~ 1 s	400kPa to 730 kPa	-	O (Thermochromic)	Resistance	10
	SWNT/PDMS	~ 10 s	< 200 kPa	-	O (Electrochromic)	Resistance	11
	Zns:Cu/PDMS	~ 100 ms	< 25 kPa	11.63 kPa^{-1}	O (ACEL)	Capacitance	Our work

Type	Active materials	Resolution	Sensing range	Sensitivity	Display (device or material)	Functionality	Ref.
Temperature	CNT/PEDOT:PSS	-	25.8-53.2 ℃	(0.25-0.63) % $℃^{-1}$	X	Flexible	12
	SWCNT/self-healing polymer	1 ℃	0-80 ℃	-	X	Stretchable/self-healable	13
	Graphene/PDMS	-	30-100 ℃	(1.05-2.11) % $℃^{-1}$	X	Stretchable	14
	rGO/P(VDF-TrFE)	0.1 ℃	30-80 ℃	-	X	Flexible	15
	ZnS:Cu/PDMS	-	20-100 ℃	0.49 % $℃^{-1}$	O (ACEL)	Flexible	Our work

Type	Active materials	Analyte	Sensing range	Sensitivity	Display (device or material)	Functionality	Ref.
Sweat	CNT/PU	NH_4^+, glucose	(0.1-100) x 10^{-3} M (NH_4^+)<br>(0-10) x 10^{-3} M (glucose)	-	X	Stretchable	16
	Graphene hybrid	Glucose	(0.01-0.7) x 10^{-3} M	1 μA mM^{-1}	X	Stretchable	17
	CNT/PU	Na^+, K^+	(0.1-100) x 10^{-3} M	59.4 mV $log[Na^+]^{-1}$<br>56.5 mV $log[K^+]^{-1}$	X	Textile-based stretchable	18
	Carbon ink	Zinc	(0.1-2.0) μg mL^{-1}	23.8 μA mL $μg^{-1}$	X	Tattoo	19
	ZnS:Cu/PDMS	Na^+	(10-160) x 10^{-3} M	0.19 % mM^{-1}	O (ACEL)	Flexible	Our work

Supplementary Table S1. The characteristics of pressure, temperature and sweat sensors recently reported in the literature for comparison with a single, multifunctional ISDEE.

Supporting videos

Video S1. Monitoring the bending motion as a function of bending angle with an ISDEE

Video S2. Imaging a fingerprint in addition to capacitance sensing with an ISDEE

References

[1] S. Park, H. Kim, M. Vosgueritchian, S. Cheon, H. Kim, J. H. Koo, T. R. Kim, S. Lee, G. Schwartz, H. Chang, Z. Bao, *Adv. Mater.* **2014**, *26*, 7324.

[2] S. Yao, Y. Zhu, *Nanoscale* **2014**, *6*, 2345.

[3] J. Lee, H. Kwon, J. Seo, S. Shin, J. H. Koo, C. Pang, S. Son, J. H. Kim, Y. H. Jang, D. E. Kim, T. Lee, *Adv. Mater.* **2015**, *27*, 2433.

[4] S. W. Lee, S. H. Cho, H. S. Kang, G. Kim, J. S. Kim, B. Jeong, E. H. Kim, S. Yu, I. Hwang, H. Han, T. H. Park, S. H. Jung, J. K. Lee, W. Shim, C. Park, *ACS Appl. Mater. Interfaces* **2018**, *10*, 13757.

[5] X. Wang, Y. Gu, Z. Xiong, Z. Cui, T. Zhang, *Adv. Mater.* **2014**, *26*, 1336.

[6] J. Park, Y. Lee, J. Hong, M. Ha, Y. Do Jung, H. Lim, S. Y. Kim, H. Ko, *ACS Nano* **2014**, *8*, 4689.

[7] H. Bin Yao, J. Ge, C. F. Wang, X. Wang, W. Hu, Z. J. Zheng, Y. Ni, S. H. Yu, *Adv. Mater.* **2013**, *25*, 6692.

[8] C. Wang, D. Hwang, Z. Yu, K. Takei, J. Park, T. Chen, B. Ma, A. Javey, *Nat. Mater.* **2013**, *12*, 899.

[9] S. Y. Chou, R. Ma, Y. Li, F. Zhao, K. Tong, Z. Yu, Q. Pei, *ACS Nano* **2017**, *11*, 11368.

[10] G. Kim, S. Cho, K. Chang, W. S. Kim, H. Kang, S.-P. Ryu, J. Myoung, J. Park, C. Park, W. Shim, *Adv. Mater.* **2017**, *29*, 1606120.

[11] H. H. Chou, A. Nguyen, A. Chortos, J. W. F. To, C. Lu, J. Mei, T. Kurosawa, W. G. Bae, J. B. H. Tok, Z. Bao, *Nat. Commun.* **2015**, *6*, 8011.

[12] S. Harada, K. Kanao, Y. Yamamoto, T. Arie, S. Akita, K. Takei, *ACS Nano* **2014**, *8*, 12851.

[13] H. Yang, D. Qi, Z. Liu, B. K. Chandran, T. Wang, J. Yu, X. Chen, *Adv. Mater.* **2016**, *28*, 9175.

[14] C. Yan, J. Wang, P. S. Lee, *ACS Nano* **2015**, *9*, 2130.

[15] T. Q. Trung, S. Ramasundaram, S. W. Hong, N. E. Lee, *Adv. Funct. Mater.* **2014**, *24*, 3438.

[16] A. J. Bandodkar, I. Jeerapan, J. M. You, R. Nuñez-Flores, J. Wang, *Nano Lett.* **2016**, *16*, 721.

[17] H. Lee, T. K. Choi, Y. B. Lee, H. R. Cho, R. Ghaffari, L. Wang, H. J. Choi, T. D. Chung, N. Lu, T. Hyeon, S. H. Choi, D. H. Kim, *Nat. Nanotechnol.* **2016**, *11*, 566.

[18] M. Parrilla, R. Cánovas, I. Jeerapan, F. J. Andrade, J. Wang, *Adv. Healthc. Mater.* **2016**, *5*, 996.

[19] J. Kim, W. R. De Araujo, I. A. Samek, A. J. Bandodkar, W. Jia, B. Brunetti, T. R. L. C. Paixão, J. Wang, *Electrochem. commun.* **2015**, *51*, 41.
